# Supplementary material for: Cholesterol accessibility at the ciliary membrane controls hedgehog signaling
Source: eLife. 2019 Oct 30;8:e50051. doi: 10.7554/eLife.50051 (PMC6850779; doi:10.7554/eLife.50051)
Supplement: Supplementary file 6. — This file describes reagents used in this study, including (when available or applicable) the type of reagent, the designation, the source and the catalogue numbers. [file elife-50051-supp6.docx]

| **Key Resources Table** | | | | |
| --- | --- | --- | --- | --- |
| **Reagent type (species) or resource** | **Designation** | **Source or reference** | **Identifiers** | **Additional information** |
| strain, strain background (*Escherichia coli*) | BL21 Rosetta DE3 pLYS | Millipore Sigma | Cat# 71403-3 |  |
| cell line (*Mus musculus*) | NIH/3T3 Flp-In | Thermo Fisher Scientific | Cat# R76107 |  |
| cell line (*Mus musculus*) | NIH/3T3 Flp-In, *Dhcr7-*/- | This paper |  | Cell line maintained in R. Rohatgi lab |
| cell line (*Mus musculus*) | NIH/3T3 Flp-In, *Dhcr24-*/- | This paper |  | Cell line maintained in R. Rohatgi lab |
| cell line (*Mus musculus*) | NIH/3T3 Flp-In, *Lss-*/- | This paper |  | Cell line maintained in R. Rohatgi lab |
| cell line (*Mus musculus*) | NIH/3T3 Flp-In stably expressing ARL13B-GFP | This paper |  | Cell line maintained in R. Rohatgi lab |
| cell line (*Mus musculus*) | NIH/3T3 CAS9, GLI1-GFP | Pusapati et al., 2018 |  | Cell line maintained in R. Rohatgi lab |
| cell line (*Mus musculus*) | NIH/3T3 8xGLI1 reporter | Pusapati et al., 2018 |  | Cell line maintained in R. Rohatgi lab |
| cell line (*Mus musculus*) | HM1 mESC GBS-Venus and Olig2-mKate | Pusapati et al., 2018 |  | Cell line maintained in R. Rohatgi lab |
| cell line (*Mus musculus*) | MEFS, *Smo*-/- | Luchetti, et al. 2016 |  | Cell line maintained in R. Rohatgi lab |
| cell line (*Mus musculus*) | MEFS, *Smo*-/- stably expressing WT SMO | Luchetti, et al. 2016 |  | Cell line maintained in R. Rohatgi lab |
| cell line (*Mus musculus*) | MEFS, *Smo*-/- stably expressing SMO D477G | Luchetti, et al. 2016 |  | Cell line maintained in R. Rohatgi lab |
| cell line (*Mus musculus*) | MEFS, *Smo*-/- stably expressing SMO D99A/Y134F | Luchetti, et al. 2016 |  | Cell line maintained in R. Rohatgi lab |
| cell line (*Mus musculus*) | NIH/3T3 Flp-In, GPR161-YFP | Pusapati et al., 2018 |  | Cell line maintained in R. Rohatgi lab |
| cell line (*Mus musculus*) | NIH/3T3 Flp-In, PTCH1-YFP | Rohatgi, Milenkovic et al. 2007 |  | Cell line maintained in R. Rohatgi lab |
| cell line (*Mus musculus*) | MEF, *Ptch*-/- stably expressing ARL13B-GFP | This paper |  | Cell line maintained in R. Rohatgi lab |
| antibody | anti-GLI1 (Mouse monoclonal) | Cell Signalling | Cat# 2643; RRID: AB_2294746 | WB (1:500) |
| antibody | anti-GLI3  (Goat polyclonal) | R and D Systems | Cat# AF3690; RRID: AB_2232499 | WB (1:200) |
| antibody | anti-PTCH1  (Rabbit polyclonal) | Rohatgi et al., 2007 |  | WB (1:500) |
| antibody | anti-SMO (Rabbit polyclonal) | Rohatgi et al., 2007 and Milenkovic et al., 2009 |  | WB (1:2000) |
| antibody | anti-SUFU (Rabbit polyclonal) | Humke et al., 2010 |  | WB (1:2000) |
| antibody | anti-P38  (Rabbit polyclonal) | Abcam | Cat# ab7952; RRID: AB_306166 | WB (1:2000) |
| antibody | anti-GFP  (Goat polyclonal) | Rockland | Cat# 600-101-215  RRID:  AB_218182 | IF (1:500) |
| secondary antibody | Anti-Mouse IgG (H+L)  (Peroxidase AffiniPure Donkey) | Jackson ImmunoResearch Laboratories | Cat# 715-035-150; RRID: AB_2340770 | WB (1:10,000) |
| secondary antibody | Anti-Rabbit IgG (H+L) (Peroxidase AffiniPure Donkey) | Jackson ImmunoResearch Laboratories | Cat# 111-035-144; RRID: AB_2307391 | WB (1:10,000) |
| secondary antibody | Anti-Goat IgG (H+L) (Peroxidase AffiniPure Donkey) | Jackson ImmunoResearch Laboratories | Cat# 705-035-003; RRID: AB_2340390 | WB (1:2500) |
| recombinant DNA reagent | OlyA WT (plasmid) | Endapally S. et al, 2019 |  |  |
| recombinant DNA reagent | OlyA E69A (plasmid) | Endapally S. et al, 2019 |  |  |
| recombinant DNA reagent | PFO* (plasmid) | Das A. et al, 2013 |  |  |
| sequence-based reagent | Lipid Gene CRISPR library | This paper |  |  |
| sequence-based reagent | mDhcr7 | This paper | Genomic DNA PCR primer | CAAGGGTGAGAAGCAACACA |
| sequence-based reagent | mDhcr7 | This paper | Genomic DNA PCR primer | CTCCTAGGAAGCAGCTACGG |
| sequence-based reagent | mDhcr24 | This paper | Genomic DNA PCR primer | CTCCTGAGCTAGTTATCC |
| sequence-based reagent | mDhcr24 | This paper | Genomic DNA PCR primer | CCTATCTTAGCAGCCCCG |
| sequence-based reagent | mLss, genomic DNA sequencing | This paper | Genomic DNA PCR primer | GGATTGCAGGCATCCCACC |
| sequence-based reagent | mLss, genomic DNA sequencing | This paper | Genomic DNA PCR primer | GATGCACTTTCAGTACCC |
| sequence-based reagent | mDhcr7, guide 1 Forward | This paper | CRISPR guide oligonucleotide | CACCGAGAACTCTGGTTGGATCACC |
| sequence-based reagent | mDhcr7, guide 1 Reverse | This paper | CRISPR guide oligonucleotide | AAACGGTGATCCAACCAGAGTTCTC |
| sequence-based reagent | mDhcr7, guide 2 Forward | This paper | CRISPR guide oligonucleotide | CACCGGATGGTACATACTTCCCCTC |
| sequence-based reagent | mDhcr7, guide 2 Reverse | This paper | CRISPR guide oligonucleotide | AAACGAGGGGAAGTATTGTACCATCC |
| sequence-based reagent | mDhcr24, guide 1 Forward | This paper | CRISPR guide oligonucleotide | CACCGTACGAGCTGATCCTGGCAGA |
| sequence-based reagent | mDhcr24, guide 1 Reverse | This paper | CRISPR guide oligonucleotide | AAACTCTGCCAGGATCAGCTCGTAC |
| sequence-based reagent | mDhcr24, guide 2 Forward | This paper | CRISPR guide oligonucleotide | CACCGTAGGAAGCTAAAGCTCAGAG |
| sequence-based reagent | mDhcr24, guide 2 Reverse | This paper | CRISPR guide oligonucleotide | AAACCTCTGAGCTTTAGCTTCCTAC |
| sequence-based reagent | mLss, guide 1 Forward | This paper | CRISPR guide oligonucleotide | CACCGCCACCGATCTCACCCGCTGG |
| sequence-based reagent | mLss, guide 1 Reverse | This paper | CRISPR guide oligonucleotide | AAACCCAGCGGGTGAGATCGGTGGC |
| sequence-based reagent | mLss, guide 2 Forward | This paper | CRISPR guide oligonucleotide | CACCGTGTGGGTATAGTGTGATATG |
| sequence-based reagent | mLss, guide 2 Reverse | This paper | CRISPR guide oligonucleotide | AAACCATATCACACTATACCCACAC |
| peptide, recombinant protein | Atto-647 maleimide | Sigma | Cat# 05316-1MG-F |  |
| peptide, recombinant protein | Alexa Fluor 647 NHS ester | Life Technologies | Cat# A-20006 |  |
| peptide, recombinant protein | Sonic Hedgehog | Bishop et al., 2009 |  |  |
| peptide, recombinant protein | bFGF | R&D Systems | Cat# 3139-FB-025 |  |
| commercial assay or kit | *Power* SYBR Green Cells-to-CT Kit | Thermo Fisher Scientific | Cat# 4402955 |  |
| commercial assay or kit | Qubit dsDNA HS Assay Kits | Thermo Fisher Scientific | Cat# Q32854 |  |
| commercial assay or kit | PureLink Genomic DNA Mini Kit | Thermo Fisher Scientific | Cat# K1820-00 |  |
| chemical compound, drug | cholesterol | Sigma | Cat# C8667-5G |  |
| chemical compound, drug | methyl beta cyclodextrin | Sigma | Cat# C4555-5G |  |
| chemical compound, drug | Myriocin | Cayman Chemicals | Cat# 35891-70-4 |  |
| chemical compound, drug | Fumonisin B1 | Cayman Chemicals | Cat# 116355-83-0 |  |
| chemical compound, drug | Egg sphingomyelin | Avanti Polar Lipids | Cat# 860061P |  |
| chemical compound, drug | Staurosporine | Millipore Sigma | Cat# 19-123 |  |
| chemical compound, drug | Vismodegib | LC Labs | Cat# V-4050 |  |
| chemical compound, drug | Buttermilk sphingomyelin | Matreya LLC | Cat# 1329 |  |
| chemical compound, drug | phosphatidylcholine | Avanti Polar Lipids | Cat# 840051P |  |
| chemical compound, drug | CHIR99021 | Axon Medchem LLC | Cat# Axon 1386 |  |
| chemical compound, drug | Retinoic Acid | Sigma-Aldrich | Cat# R2625 |  |
| chemical compound, drug | U18666A | Douglas Covey Lab |  |  |
| chemical compound, drug | SAG | SelleckChem | Cat# S7779 |  |
| chemical compound, drug | Dimethyl Sulfoxide | Fisher | Cat# 9224-06 |  |
| chemical compound, drug | 2-mercaptoethanol | Gibco | Cat# 21985023 |  |
| chemical compound, drug | fatty acid free bovine serum albumin | Sigma | Cat# A8806-5G |  |
| chemical compound, drug | Dulbecco’s Modified Eagle’s Medium | Fisher | Cat# SH30081FS |  |
| chemical compound, drug | Fetal bovine serum | Sigma-Aldrich | Cat# S11150 |  |
| chemical compound, drug | Sodium pyruvate | Gibco | Cat# 11-360-070 |  |
| chemical compound, drug | L-glutamine | Gemini Bio-products | Cat# 400106 |  |
| chemical compound, drug | penicillin/ streptomycin | Gemini Bio-products | Cat# 400109 |  |
| chemical compound, drug | Nonessential amino acids | Gibco | Cat# 11140076 |  |
| chemical compound, drug | Optimem | Fisher | Cat# 31985-070 |  |
| chemical compound, drug | Lipoprotein depleted serum media | Kalen Biomedical, LLC | Cat# 880100-2 |  |
| chemical compound, drug | Dulbecco’s Modified Eagle’s Medium F12 | Fisher | Cat# 21331020 |  |
| chemical compound, drug | Neurobasal Medium | Gibco | Cat# 21-103-049 |  |
| chemical compound, drug | N-2 Supplement | Fisher | Cat# 17502001 |  |
| chemical compound, drug | B-27 Supplement | Thermo Fisher Scientific | Cat# 17504044 |  |
| chemical compound, drug | Albumin from bovine serum | Sigma | Cat# A3059-100G |  |
| chemical compound, drug | Matrigel (Corning 356234) | Fisher | Cat# CB-40234A |  |
| other | Corning CellBIND plates | Fisher | Cat# 08-757-214 |  |
